# Enhanced relapse prevention for bipolar disorder: a qualitative investigation of value perceived for service users and care coordinators

**DOI:** 10.1186/1748-5908-4-4

**Published:** 2009-02-09

**Authors:** Eleanor Pontin, Sarah Peters, Fiona Lobban, Anne Rogers, Richard K Morriss

**Affiliations:** 1 School of Population, Community and Behavioural Science, Faculty of Medicine, University of Liverpool, Liverpool, UK; 2 School of Psychological Sciences, Faculty of Medical and Human Sciences, University of Manchester, Manchester, UK; 3 Spectrum Centre for Mental Health Research Institute of Health Research, University of Lancaster, Lancaster, UK; 4 Health Care National Primary Care Research and Development Centre, University of Manchester, Manchester, UK; 5 School of Community Health Sciences, Faculty of Medicine and Health Sciences, University of Nottingham, Nottingham, UK

## Abstract

**Background:**

Enhanced relapse prevention (ERP) is a psychological intervention delivered by mental health professionals to help individuals with bipolar disorder (BD) recognise and manage early warning signs for mania and depression. ERP has an emerging evidence base and is recommended as good practice for mental health professionals. However, without highly perceived value to both those receiving (services users) or delivering it (health professionals), implementation will not occur. The aim of this study is to determine what values of ERP are perceived by service users (SUs) and mental health professionals (care coordinators, CCs) providing community case management.

**Methods:**

A nested qualitative study design was employed as part of a randomised controlled trial of ERP. Semi-structured interviews were conducted with a purposive sub-sample of 21 CCs and 21 SUs, and an iterative approach used to develop a framework of conceptual categories that was applied systematically to the data.

**Results:**

The process of implementing and receiving ERP was valued by both SUs and CCs for three similar sets of reasons: improved understanding of BD (where a knowledge deficit of BD was perceived), enhanced working relationships, and improved ways of managing the condition. There were some differences in the implications these had for both CCs and SUs who also held some reservations.

**Conclusion:**

CCs and SUs perceive similar value in early warning signs interventions to prevent relapse, and these have particular benefits to them. If this perceived value is maintained, CCs and SUs in routine practice may use ERP long-term.

## Background

Bipolar disorder (BD) is a serious mental illness affecting one to two percent of the population [1], characterised by separate periods of mania (elated mood, disinhibited behaviour, overactivity, inflated self-esteem, decreased need for sleep) and depression (low mood, profound loss of interest, changes in sleep and appetite, low self-worth, suicidal ideas and plans). BD is a lifelong condition with a peak onset at 19 years of age [2]. Most patients diagnosed with BD will have contact with secondary care mental health services at some point in their lives, but only patients with recent episodes of illness are in continuing care from such services [1]. In the United Kingdom (UK), continuing care for BD is provided by community mental health teams or outpatient care in mental health services.

Surveys of patient organisations across Europe and the US reveal there is a strong desire by patients with BD for both self-help and psychological treatments in addition to pharmacotherapy [3,4]. Recent national treatment guidelines recommend that structured psychological interventions should be offered to people with BD [5-7].

One form of psychological intervention is relapse prevention (RP), which (taking a psychoeducation approach) teaches people with BD to recognise early warning signs to manic and depressive episodes [8,9]. This particular intervention is one of a number of psychosocial interventions recommended in national guidelines [5,6], but currently it is not widely implemented in routine clinical practice. Prospective and retrospective studies show that people with BD recognise a pattern of symptoms and signs before each manic or depressive episode, idiosyncratic to the type of episode and to each person [10]. RP interventions are effective in increasing time to relapse, reducing the percentage of people hospitalised, and improving social functioning [11], although the effectiveness against depressive episodes may be confined to interventions incorporating other psychological techniques [11,12]. The effectiveness of interventions involving early warning signs and psychoeducation incorporated in a system of collaborative care may last several years, and so may be cost effective [12,13].

The main advantage of RP interventions compared to more sophisticated approaches involving early warning signs, such as some forms of cognitive behaviour therapy and family therapy [14,15], is that simple RP interventions can be taught more quickly and easily to both health professionals and patients, and do not rely on an extensive training in psychotherapy [9,12,13]. Moreover, psychotherapists rarely have experience in working with BD. Potentially, RP would be much easier to implement on a large scale in everyday clinical practice than interventions requiring specialist psychotherapy skills. However, in many health care systems, such as the National Health Service (NHS) in the UK, there is no established means of service delivery charged with the responsibility of delivering RP interventions or indeed any other psychosocial interventions. As well as lack of availability of treatment, routine services may not deliver psychological interventions as effectively as the authors of the treatment who conducted the original research. As a result, service delivery of known effective interventions, even when supported by national or state guidelines, may be haphazard [16,17].

To investigate whether generalisable implementation was possible using routinely available community mental health services, Lobban *et al*. [18] devised an enhanced form of RP to be used alongside other interventions such as pharmacotherapy (see Table 1). Enhanced Relapse Prevention (ERP) is offered by care coordinators (CCs) who are psychiatric nurses, social workers, or occupational therapists practising case management [19] from community work bases (Community Mental Health Teams or CMHTs) alongside psychiatrists and clinical psychologists in the UK NHS. A similar system for providing community follow-up care for people with serious mental illness is found in many services across the world but not universally [4,19,20]. The ERP intervention is accompanied by an easy-to-use manual outlining each of the six, sixty-minute sessions, copies of which are read by service users (SUs), relatives, and CCs [18].

**Table 1:** Features of Enhanced Relapse Prevention [18]

Key elements of the ERP intervention as explicitly recommended in the NICE guidelines for Bipolar Disorder (NICE 2006). Carried out separately for depression, mania and mixed episodes, they include:
• Psychoeducation
• Developing detailed analysis of previous episodes
• Identifying trigger situations and early warning signs
• Enhancing coping strategies for mood changes
• Negotiating an action plan for responding to early warning signs
• Agreeing with clinical services about how they will respond to different stages of relapse

The aim of this study is to determine the value of an ERP intervention, as perceived by SUs and their CCs. The use of any health intervention, especially psychological interventions that require effort to change routine practice, depends on whether the perceived benefits of the intervention outweigh perceived barriers [21], and this applies as much to health professionals as recipients of health care [22]. If interventions are seen to be helpful in staying well, they may well be used longer term by SUs [23] and CCs. If an intervention has no or limited perceived value to either health professionals or SUs, it will probably not be used beyond the training period and will almost certainly be ineffective as a clinical service.

## Methods

As this was the first study of the perceived values of ERP, a qualitative approach was selected since it required no previous assumptions to be made. Value is a subjective experience that is not usually measured, as quantitative instruments that are psychometrically sound and sensitive to change have yet to be developed.

### Sampling

The qualitative investigation was nested within a cluster randomised control trial to assess the feasibility of training of CCs to offer ERP for BD [18]. Ethical approval was obtained through the Central Office for Research Ethics Committees. Ninety-six SUs and 112 CCs from 23 Community Mental Health Teams (CMHTs) in the northwest of England were recruited for the trial and formed the strategic sampling pool. Further details of recruitment to the trial are reported elsewhere [18]. CCs were randomly allocated by CMHT to receive training in ERP or to continue to offer treatment as usual.

Purposive sampling was used to select participants for interviews to ensure a full range of views were represented. All interviews were conducted within 12 months of the delivery of the intervention. Out of 96, 21 SUs were selected on the basis of whether or not they had a relapse since baseline and time since diagnosis. In addition, 21 CCs were selected (out of a possible 112), on the basis of how many clients they had trained in ERP and their occupational background. Participants who had received or had delivered the ERP intervention and those who were in the control group,' treatment as usual' (TAU) of the trial were included. Those who had not received or delivered ERP were interviewed to enable sufficient conclusions to be made about the context in which ERP is delivered. Semi-structured interviews were conducted until data saturation was achieved. All those approached agreed to be interviewed. The final sample comprised 21 SUs and 21 CCs (see Tables 2 and 3).

**Table 2:** Summary clinical and demographic information of care coordinators interviewed (n = 21)

**Characteristic**	**n (%)**
Group	
enhanced relapse prevention	14 (67)
treatment as usual	7 (33)

Sex	
female	14 (67)
male	7 (33)

Age(years)	mean 45 (range 29–57)

Professional background	
community psychiatric nurse	18 (86)
occupational therapist	2 (10)
social worker	1 (4)

Years worked in community mental health team	mean 7.2 (range 1–30)

Deprivation indices of work place**	

lower quartile (least deprived)	3 (21.5)
Mid lower quartile	3 (21.5)
Mid upper quartile	1 (7)
upper quartile (most deprived)	7 (50)

Caseload balance	

number SUs with BD diagnosis	mean 6 (range 1–9)
% of caseload with BD diagnosis	mean 20% (range 3–40%)

number of SU receiving intervention*	mean 2 (range 0–3)

*At time of interview. ERP group only

** Townsend deprivation scores are the best indicator of material deprivation and disadvantage currently available in England. Postcodes were converted into Townsend deprivation indices (Townsend 1998) and categorised into bands in accordance with deprivation indices for England.

**Table 3:** Summary clinical and demographic information of service users interviewed (n = 21)

**Characteristic**	**n (%)**
Group	
enhanced relapse prevention	14 (67)
treatment as usual	7 (33)

Sex	
female	13 (62)
male	8 (38)

Age (years)	mean 47 (range 24–63)

Deprivation indices of work place**	
lower quartile (least deprived)	3 (14)
mid lower quartile	4 (19)
mid upper quartile	6 (29)
upper quartile (most deprived)	8 (38)

Employment status	
unemployed	10
part or full-time employed	7
retired	2
student	2

No. of previous episodes	
Depression (n = 21)	
0–2	5 (24)
3–5	0 (0)
6–10	1 (5)
11–20	4 (19)
>20	5 (23)
unknown	6 (29)
Mania (n = 21)	
0–2	3 (14)
3–5	4 (19)
6–10	2 (11)
11–20	3 (14)
>20	4 (19)
unknown	5 (23)

Years since first episode	mean 21 (range 1–46)

Relapsed since ERP intervention*	
Enhanced Relapse prevention	
Yes	6 (43)
No	8 (57)
Treatment as Usual	
Yes	2 (29)
No	5 (71)

*At time of interview

** Townsend deprivation scores are the best indicator of material deprivation and disadvantage currently available in England. Postcodes were converted into Townsend deprivation indices (Townsend 1998) and categorised into bands in accordance with deprivation indices for England.

### Procedure

Participants were interviewed by a researcher (EP). SUs were interviewed in their own homes and CCs in their place of work. Interviews with SUs averaged 60 minutes (range 15 to 120 minutes) and with CCs 45 minutes (range 25 to 96 minutes). All participants gave written informed consent. In order to prevent the researcher from being perceived as part of the ERP training intervention, the qualitative research team was not located in the trial office, and the researcher was employed by a different funding body. Independence from the trial team was considered important for increasing the data quality. It was made clear to SUs and relatives that the interviewer was not involved with or monitoring their care team. Similarly, it was made clear to CCs that the researchers were not part of the ERP training team and not monitoring whether they had delivered ERP correctly. SUs were asked to talk about their experiences of taking part in the ERP intervention and of the services they received from their CC and community mental health team. CCs were also asked to recount their experiences of delivering the ERP intervention, and about their role in working with SUs. Interviews were structured around a topic guide but the researcher was responsive to issues emerging from participants' accounts. Emerging themes were explored throughout the data collection process and specifically attended to and developed in further interviews in an iterative process. All interviews were digitally recorded and transcribed verbatim.

### Analysis

A grounded theorizing [24] approach was used to develop conceptual categories from the data by thematic analysis. Categories and memos were coded into a document that was refined and elaborated in light of incoming data and analysis. Authors EP and SP identified recurrent patterns, testing and modifying them by constant comparison, 'cycling' between sets of data and the developing account of them and by discussion. All interviews were separately analysed by EP and read by at least one other researcher [SP, FL, AR, RM] to check for reliability. Periodically, the developing thematic analysis was referred to the broader group of those involved in trial, which included researchers and clinicians from clinical and health psychology, psychiatry, medical sociology, and nursing. Triangulation is a recognised method to increase the trustworthiness of data analysis [25]. This was achieved in two ways: first, through data triangulation whereby both the perspectives of SU and CC were explored and categorised as themes in the final analysis if they were independently identified by both groups; and second, by investigator triangulation, whereby the analytical framework was developed by authors from different theoretical and discipline perspectives. Additional standards employed to assess the developing analysis were coherence and 'theoretical validity' whereby conclusions should connect with theoretical ideas beyond the present study [26]. Furthermore, we were also concerned with the 'catalytic validity' of the findings, that is, they should not merely describe, but should have the potential to change clinical practice or research [27].

## Results

The process by which ERP functions and is valuable for SUs and CCs were categorised into three main themes: elaborated understanding of BD, enhanced working relationships, and developed ways of working with and managing BD. The meaning and implication for each theme differed slightly for each participant group (see Table 4). For each, the three themes impact on one another and are interrelated.

**Table 4:** Value and clinical implications of ERP reported by care coordinators (CC) and service users (SU)

**Value**	**Implications for CC**	**Implications for SU**
Improved Understanding of Bipolar Disorder	• Learns about BD	• Learns about BD
	• Learns about early warning signs, triggers and coping strategies	• Learns about early warning signs, triggers and coping strategies
	• Acquires new skills for working with individuals with BD – increases competence and confidence of working with individuals with BD	• Increases acceptance of diagnosis and rationale for medication concordance
	• Acquires new skills and strategies that generalise to working with individuals with other disorders	• Reduces feeling of isolation and fear of BD
	• Gains further understanding of SU perspective and experience of BD	• Allows opportunity to reflect and make sense of lives
	• Need to manage SUs distress and anxiety talking about past illness episodes	• Distress and anxiety talking about past illness episodes
		
Developed ways of working with and managing Bipolar Disorder	• More contact with SU	• More contact with CC
	• Opportunity to work with SU when well	• Improves recognition of triggers, early warning signs and coping strategies
	• Added burden to workload and time	• Increases monitoring of mood and behaviour
	• Increases complexity of role	• Empowerment and control over BD
	• Sessions are more structured and focused	• Identifying and using coping strategies to prevent relapse
	• Provides added sense of purpose	• Creation of concise, individualised action plan
	• Documentation to support working	• Relapses can occur too quickly to use action plan
	• Creation of concise, individualised action plan	• SU not motivated to prevent mania relapse
	• Concerns that action plan not used in crisis by SU and wider team	
	• Identifying and reinforcing personalised coping strategies	
		
Enhanced Working Relationships	• Discovers new relevant information	• Shares new relevant information
	• Collaborative working	• Collaborative working
	• Is considered as more trustworthy	• Increases trust in CC
	• Improves contact by SU when needed	• Improves contact with services when needed
	• Increased dependency on CC rather than service as a whole	• Increased dependency on CC rather than service as a whole
	• Changes relationship dynamic	• Changes relationship dynamic

### Elaborated understanding of Bipolar Disorder

This theme refers to the information, understanding, perception, and awareness SUs and CCs have about BD, and more specifically its impact on SUs' life and circumstances.

The ERP intervention increased perceived knowledge of BD for both SUs and CCs. CCs perceived their previous knowledge was limited and few had received any formal training specific to BD. This was striking given that the average length of time worked in community mental health team was seven years, and the proportion of individuals with BD in CC caseloads was reported to be up to 40% (see Table 3). Value was placed on the six-session training given to CCs prior to commencement of implementing the intervention because it seemingly filled a knowledge deficit. CCs acquired new knowledge about BD and as a consequence felt their confidence and ability to work with patients had increased:

I said, 'do you know what bipolar is?' And she [said] 'no one has ever explained it'. So we sat and we went through it, and now she fully accepts that she has got bipolar . . . it has [given] me that much confidence to be able to do that. (63: CC, ERP)

ERP had generalisable value, because CCs viewed ERP principles and techniques as new elements of a 'tool kit' that could be used with other client groups. CCs reported that through increased understanding of BD their awareness of relapse triggers and early warning signs had improved. This was the mechanism by which they considered ERP could impact on relapse:

It might reduce the severity, maybe, because I'll be more aware as well, and other people around are more aware and can sort of pick up on things earlier (11: CC, ERP)

Discussing this information with SUs also provided an opportunity for increased understanding and appreciation of the patient perspective:

You work with people for several years, but when you do something like this ERP it gives you more understanding of the symptoms and the difficulties that they have had (28: CC, ERP)

Furthermore, new information about the SU and their family was disclosed, which further increased their understanding of the context and experience of BD:

I do know quite a bit more about certainly what is going on in the family and the family dynamics, so there is a bit more knowledge there . . . which she probably wouldn't have maybe told me (14: CC, ERP)

Acquiring a better understanding of BD was also valuable to SUs. To some extent this was achieved through simply gaining new information:

I learnt about the illness. I didn't know there were all different parts to the illness you know. I didn't know, like mania, depression, they were all combined, so I learnt a lot (14: SU, ERP)

As a consequence, some SUs found this helped them accept their diagnosis and recognise the role of medication in relapse prevention:

I learnt about the illness... I realised I have got bipolar, only when I have done this [ERP]. Because I thought I could stop taking my tablets and I can't (9: SU, ERP)

It also provided an argument for why SUs should respond to any warning signs:

Knowledge is power, isn't it? . . . If they are beginning to go into a relapse, the wish is to stick their head in the sand . . .'I will be alright by the end of the week, this is just passing'. Whereas what this work [ERP] was doing is saying you feel it – well react! Straight off, because we have only got limited time (39: CC, ERP)

An element that some SUs identified as valuable was feeling they could now 'face' the illness without fear, realising they are not alone in suffering from BD:

At first, like before I had the intervention, it was like, I just felt like I was the only one out there . . . It was just learning, really. Being educated about it, whereas before all I knew about it was . . . That's all I knew about it (9: SU, ERP)

An element of the intervention that was particularly valued was creating a timeline, charting past manic and depressive episodes. This gave the opportunity to make sense of what had occurred in their lives, to reflect on the past and understand how the illness had affected them as an individual. While the value of this process was recognised, some SUs found recalling past episodes uncomfortable, especially identifying triggers and warning signs of depression:

She finds it painful looking at when she was being depressed . . . Once she is out of that depression she doesn't want to revisit there (28: CC, ERP)

SUs described talking about their illness as emotionally tiring and upsetting, and some reported induced feelings of anxiety. CCs were aware of this being demanding, and one reported that looking at triggers and early warning signs had the potential to induce a relapse.

### Developed ways of working with and managing Bipolar Disorder

For CCs, managing BD meant adjusting the ways they worked with clients. Management for SUs involved self-care, including their use of health services and advice from CCs. Both sets of participants described differently the changes to how they managed BD.

### Working with service users with bipolar disorder

ERP meant CCs spent more time with SUs, because sessions were longer or more frequent than usual – particularly when SUs were well, rather than (as is usual), having most contact during a crisis or relapse. Although spending more time together was valued, CCs perceived this as an added burden on their workload and time. Progress could be hampered by competing demands from SUs, which required them to react to current problems rather than engaging in preventative therapy. CCs described having to juggle two aspects of their role – reaction (crisis management, solving practical problems, advocacy and triage) and prevention (advice, motivation, documenting care plans). For some, the intervention was recognised as building on this latter role. For others, this was a less familiar role and necessitated a change in their way of working, which increased the complexity of their role, and hence was demanding. Some reported that ERP sessions diverted being able to discuss other issues of importance to SUs, such as accommodation or financial concerns:

The client has got other issues going on, and sometimes its [ERP] is just not a major . . . agenda for them (14; CC, ERP)

The time spent together doing ERP was more formal and structured compared with previous encounters or that described by untrained CCs:

Doing it this way it's very focused . . . and moves on progressively, whereas sometimes if you are not careful you can do tea-drinking exercises. You go in 'how are you today? . . . Are you taking your meds alright? . . . Sleeping and eating alright? Any pressures in your life?' . . . It can become very generalised, and I found with this it was good that you had a real focus (39: CC, ERP)

CCs valued the structure and focus of ERP as it gave them a sense of purpose:

I am actually doing something useful, rather than just talking about nothing (48: CC, ERP)

Despite the need for some planning prior to the sessions and how this also added to their workload, many reported the value of having a manual and accompanying documentation was valued. Getting plans and information down on paper gave SUs something to refer to:

They have actually got visual stuff to look at and remind them of what is happening . . . to look at and say well 'ok, if this, that is what I am going to avoid'. (6: CC, ERP)

Formulating an action plan was useful not just for the SU but for CCs and other health professionals. Care plans used in the TAU group were largely undervalued and seen as 'a waste of time' 'ineffective', 'patronising', and 'burdensome', having little function for either CCs or SUs:

I would say that 75% of my service users would not open their care plan from the day it was sent out to them to the day it's renewed (33: CC, TAU)

In contrast, the action plans devised as part of ERP were viewed as being accessible and used by SUs:

She has got it [action plan] up on her wardrobe, so when she opens her wardrobe she can see it (14: CC, ERP)

I got all the symptoms on mania and depression, I have got two sheets . . . if I find am not being able to cope with any of them, I have got the numbers to ring. (14: SU: ERP)

However this was not necessarily true for all participants: some reported only using the plan occasionally, particularly when SUs were stable.

CCs liked that the action plan was 'much more personal and appropriate to that one individual' as well as being more concise. It was useful for disseminating the plan to other health professionals in that it told them 'what to do', which was particularly important in case the SU did not have access to their CC or usual care providers during a crisis. Nevertheless, several CCs still voiced concerns that SUs would not use it in a crisis, or that it would not be accessed and used by the wider care team.

In addition to devising personalised action plans, CCs described valuing focussing on coping strategies particular to the individual:

Very specific things to them. So like this one lady, she would go and have a massage or a facial, or have her nails done. That is what she likes doing to relax, whereas that man that I saw he would go and clean his motor bike (48: CC, ERP)

Or more typically, to clarify and reinforce existing strategies:

Most people have already got coping skills. Its just either altering them because they are a little bit inappropriate, or just using the ones that they have already got but make it very clear to them that it is a good thing to do (48: CC, ERP)

CC valued the changes to the way they worked with those with BD. In addition, it was reported that the core elements of ERP could be applied to other conditions such as schizophrenia and psychosis:

Even though you have to use a different perspective a little bit, it is still the same, same process . . . whether that was schizophrenia or bipolar. (38: CC, ERP)

### Engaging with self-care strategies and working with services

Respondents described how increased awareness of triggers, early warning signs, and coping strategies had changed behaviour, particularly medication adherence. By looking back over previous episodes some believed their awareness of their early warning signs to relapse had increased:

I feel a lot more aware now of what goes on. Before I really didn't have a clue, to be honest (9; SU, ERP)

As a consequence, SUs described being better able to monitor their mood and behaviour. Moreover, instead of relying on their CC for monitoring, SUs were more able to identify early warning signs themselves:

She wouldn't have identified them as quickly. It would have been me going round and questioning her, rather than her actually identifying them herself (29: CC, ERP)

This gave a new perception of control about managing their illness:

It's like new for me, you know. You are always looking for goals . . . Not that you want to sit and feel sorry for yourself, but if you have got a focus to learn more about it then you can have more control . . . I can't be thinking 'what if I just go like that and I can't control it', which used to terrify me (21; SU, ERP)

Both SUs and CCs described this in terms of empowering for SUs:

It has actually structurally empowered the client to actually look at their own patterns of behaviour, patterns of illness, so they actually have a self-monitoring part (29; CC, ERP)

In consequence, SUs responded to early warning signs more quickly, contacting services earlier, more appropriately, and at a point where relapse prevention was possible:

When she has two nights of no sleep, she becomes ill very quickly and she is one of the people that text me to say I am not sleeping, 'can I have some tablets'? I get it arranged, she comes and she picks it up, and that's that. She hasn't had a relapse in a year and so she has been brilliant . . . And I have found it really beneficial (48: CC, ERP)

They also found other ways of responding to early warning signs to prevent them developing:

You just have to think of the steps what you have got to do, you know, the programme thing [ERP], be calm, do whatever, relax . . . have a bath and work it through, talk to your care worker, or whatever, and it's better, and that's what I did (21: SU, ERP)

However, some SUs felt coping strategies were ineffective, as relapses occurred too quickly and that there was little they could do to prevent them. For others, they chose not to intervene – one individual explained that although she could recognise the early signs of mania, because they were positive, she was unmotivated to try to prevent relapse:

I know the early signs of highs, I talk faster. I don't go to bed. I nibble; I don't eat proper meals or anything . . . I don't want to watch out for them, I want to go out and enjoy myself (18; SU, ERP)

### Enhanced working relationships

Another set of values identified by both CCs and SUs related to an improved relationship between SUs and CCs and the mental health service. Because of the increased time SU spent with their CCs, there was more time to get to know one another better:

We got on really well . . . didn't so much at the beginning, but because I hardly saw her . . . We just talk about absolutely anything, which is great. (35: SU, ERP)

It also gave CCs and SUs an opportunity to talk about things they had not necessarily discussed before. SUs and CCs independently described the way in which ERP gave an opportunity to work collaboratively. SUs talked about the value of 'having a say in it' and 'working together' with their CC. CCs talked about a 'working relationship' and ERP being a 'relationship-former'. Working collaboratively also built trust:

I have worked more closely with her, even though I was seeing her weekly; it's probably been closer because we have actually been doing something together. So yes, probably more trust. (76: CC, ERP)

Improved relationships and trust was reported to influence help-seeking behaviour and increase contact with service providers when support is needed:

And she now, more so than before, [will] ring me if there was a problem. Whereas before, even though I say to people' ring me if there is', nine times out of ten they think, well, they are being a nuisance so I won't. But that has gone, with [SU] she would. (76: CC, ERP)

However, one CC reported that the impact of increased knowledge of the SU and of an enhanced relationship created a greater dependency on the CC as an individual care provider rather than the service as a whole:

Because they feel I know a lot about them and their illness and its presentation, they feel quite anxious when I am not around, if I am on leave (46; CC, ERP)

While ERP was reported by many of the SUs and CCs to change their relationship for the better, it was recognised by some that ERP had a less positive impact on their relationship. Changing the balance of roles of CCs and their way of working with SUs had the potential to change the dynamic of their relationship whereby it became more structured, focused, and less reactive:

I wouldn't want all my sessions with [CC] to be like that [ERP] because they would be too heavy, you know (19; SU, ERP)

## Discussion

Within the context of a contemporary policy commitment to making psychological interventions more widely available within health services, the significance of findings of this study assumes significant salience. In the UK, the Improving Access to Psychological Therapies (IAPT) programme seeks to provide better access to psychological therapies for people with mental health needs (DoH 2008). A fine-grained view of the components of ERP, which was the aim and focus of this study, fits with this broader policy agenda that seeks to engage a wider group of professionals in delivering a range of psychological interventions (*e.g*., primary care mental health workers) and in developing resources for SUs that are more personalized and suited to meeting individuals needs.

This is the first study to examine the perceived value of relapse prevention from the perspectives of both those delivering the intervention (CCs) and individuals with BD who are receiving the intervention (SUs). The process of implementing and receiving ERP in a community mental health setting was valued by both SUs and CCs for similar reasons: benefiting from an increased understanding of BD, developing ways of working with and managing BD, and enhancing working relationships. This is a key step in establishing the feasibility of providing relapse prevention in this way to individuals with BD [21].

Our results resonate with the findings of two previous qualitative studies of SUs with BD. Previously, it was found that those with BD who had stayed well for two years valued being able to recognise triggers and early warning symptoms, and this was identified as a means of preventing further episodes [23]. In addition, SUs taught the life goals program – which also includes the recognition of early warning symptoms and triggers – particularly valued the interactional and collaborative nature of these interventions when the SU started to become symptomatic [29]. This echoes our findings of the value that both SUs and CCs placed on the impact ERP had on their relationship.

ERP delivered through case management in community mental health teams also fulfils an unmet need. SUs report frequent discontinuities of medical and psychological care, inadequate and inappropriate care in crises, and exclusion of carers and families from management decisions [30]. ERP provides continuity of care, defines precisely when crisis intervention is required and what should happen, and has the capability to involve caregivers and families in the management process [18]. Nearly 70 percent of SUs with BD in a large international survey wanted information about how they might prevent further episodes, their preference being to receive this information from health professionals who could intervene rather than advocacy services [4].

Although there were many similarities between the perceived value of ERP for CCs and SUs, there were some important differences in emphasis. SUs emphasised reduced isolation, empowerment, making sense of their lives, and acceptance as a result of their diagnosis and medication. Interventions that promote a bigger role of the SU in management of their condition tend to reduce social isolation and increase empowerment [31], although SUs can sometimes develop unrealistic expectations of such approaches [32]. Increased acceptance of diagnosis and medication will be welcome to most clinicians; improved adherence to lithium treatment has been demonstrated before using group psychoeducation involving RP [33].

ERP was perceived to increase individuals' understanding of BD. SUs were given knowledge that was specific to BD that fit with their personal experience of living with the disorder. It maybe therefore, that being given information that is specific enough to the individual's illness and experience is a necessary pre-requisite to promote relapse prevention. The data also revealed the lack of previous knowledge about BD that most CCs felt they had prior to undertaking the ERP training. CCs emphasised the benefits of more structure and focus to their thinking about relapse, their intervention, and their action plans as a result of ERP training. Furthermore, although ERP was developed specifically for BD, CCs spontaneously recognised that they could apply the core elements of the intervention when working with clients with other conditions. Demonstrating that an intervention can be applied to other conditions is an important selling point for its implementation.

Overall, the impression obtained of case management, as carried out in CMHTs under treatment as usual with SUs with BD, was that the skills of CCs were not being used optimally. The additional benefits of nursing intervention for SUs with BD may only arise if the CCs are properly trained and supported for the role [34]. The perceived value of ERP may be swamped by barriers to care such as lack of training and support, other work priorities, or excessive caseload size requiring CCs to prioritise reactive care over proactive care [22]. The study found evidence that ERP was a new way of working for many CCs, and these CCs could manage the increased workload and time that this new role demands. However, other CCs in other services may not cope with the extra workload and demands as well. Sufficient resources and support therefore need to be made available for CCs to implement ERP delivery so that the values of the intervention for both CCs and SUs can be maximised and maintained.

In general, ERP was valued, but not for all CCs and SUs, such as SUs who may become distressed by reviewing previous episodes of illness, who believe that they do not have the ability to prevent fast onset illness episodes [21], or value the informality of support over structured treatment. An increased dependence of SUs on individual CCs was also identified as a problem.

Case management by mental health professionals working in the community is common in the UK but it is less common, especially for younger people with BD, in other health systems [29]. In the UK and in other health systems, SUs with a case manager tend to have more severe and more recent acute bipolar episodes [20]. Further research will determine whether other groups of mental health professionals working in different systems of care, and patients with less severe and less recent bipolar episodes also value interventions such as ERP.

There are a number of limitations to the study. Rather than being able to assess actual behaviour, reliance has been on accounts of participants involved in the study. Limitations in the methodology also include the important question of whether CCs and SUs told the research team what CCs and SUs thought they wanted to hear rather than their beliefs. We tried to prevent this by presenting to CCs and SUs a separate identity for the qualitative team from the team carrying out the randomised controlled trial and the service in which the CCs worked, adopting a neutral stance in the interview process using open questions that did not indicate any preference for one treatment over another, and selecting participants who did not complete ERP as well as those who did.

In undertaking a qualitative investigation we did not seek to recruit a representative sample, but rather to access the range of available views. Participants were those who were already recruited for a training trial [18] and therefore had agreed to participate in research. Although our 100% recruitment to the qualitative study avoids the possibility of systematic bias in those interviewed compared with those in the trial, it is possible that participants hold different views to those declining to take part in the trial. Such individuals may have been less likely to see value in ERP, though it is unlikely they would have identified additional values than those identified by participants who did take part in the trial. It is possible that CCs who did not participate in the research trial may have been individuals who had the most to gain from training in psychological interventions. While we do not have any information on CCs from teams declining to take part or SUs who were eligible for ERP but were not referred by their trained CC, the strategy to include SUs who were in the TAU and CCs who did not train any clients goes some way towards addressing this point. A particular strength of the study is that interviews were conducted until thematic saturation was obtained, and data was drawn from a large and rich data set with representation from both the perspectives of CCs and SUs.

Because the study was conducted alongside a randomised controlled trial, participants were interviewed within 12 months of delivering or receiving ERP. Consequently only eight out of the fourteen SU interviewed in the ERP group had experienced a relapse by the time of the interview. It may be that over a longer period further value for SU may have been identified or elements identified early on may be less valued and this would be an interesting area for further research. Similarly for CC, experiences over a longer time period with a range of different SUs and situations may influence their views of ERP.

## Conclusion

In summary, ERP appears to be of perceived value to many CCs and SUs in the management of BD. The intervention was reported to provide a greater understanding of BD and developed ways of working with and managing BD in terms of skills and confidence in dealing with the early stages of acute episodes, and by enhancing the working relationships between CC and SU. It is recommended that SU and CC perceptions of value of an intervention (rather than just the evidence base of effectiveness) are identified and incorporated into an implementation strategy. ERP was valued by CCs and SUs; therefore, with conditions that support the introduction of complex interventions [22], its implementation could be sustained in community mental health teams in NHS secondary care mental health services. The study also has broader implications for assessing the acceptability and design of new psychosocial and self-care interventions. Rather than seeking to replace existing patient and professional practices or knowledge, interventions can usefully seek to make a link with and build on existing patient knowledge, patient self-care activities, and the working practices of health care professionals. Attention to delineating specifying and clarifying the specific roles of patients and professionals as change agents is implicated by this study, confirming previous findings that the role of patients sometimes remains implicit or ambiguous interventions about self-care [35]. Finally, introducing a new intervention to both patients and professionals allows for the mutual opportunity for consensus about the purpose of the intervention and developing a working partnership. These considerations are relevant for promoting the value of an intervention programme before it actually gets developed, tested, and implemented.

## Competing interests

The authors declare that they have no competing interests.

## Authors' contributions

All authors contributed to the design and analysis of the study. FL was principle investigator on the trial from which the participants were recruited. EP collected the data and led the analysis. SP led the writing of the paper to which all authors contributed and commented on drafts.
